# Effects of resveratrol on Th17 cell-related immune responses under tacrolimus-based immunosuppression

**DOI:** 10.1186/s12906-019-2464-1

**Published:** 2019-03-04

**Authors:** Kyoung Chan Doh, Bo-Mi Kim, Kyoung Woon Kim, Byung Ha Chung, Chul Woo Yang

**Affiliations:** 1 Convergent Research Consortium for Immunologic disease, St. Mary’s Hospital, The Catholic University of Korea, Seoul, South Korea; 2 Transplant research center, St. Mary’s Hospital, The Catholic University of Korea, Seoul, South Korea; 3 Department of Internal Medicine, College of Medicine, Seoul St. Mary’s Hospital, The Catholic University of Korea, Seoul, South Korea

**Keywords:** Th17 cells, Resveratrol, Tacrolimus, AMPK, mTOR, Organ transplantation

## Abstract

**Background:**

We previously reported that tacrolimus (Tac) does not decrease T helper 17 cells (Th17) response in kidney transplantation. In this study, we evaluated whether Resveratrol (Resv) has immunosuppressive effects by decreasing Th17 responses in Tac-based immunosuppression.

**Methods:**

We investigated the effects of Resv under Tac-treatment conditions, on CD4^+^ T cell differentiation to Th17 cells in peripheral blood mononuclear cells (PBMCs), and proliferation of CD4^+^ T cells co-cultured with human renal proximal tubular epithelial cells (HRPTEpiCs). The effects of Resv on Th17 cells were tested in the murine skin transplant model.

**Results:**

In PBMCs, Tac did not but combination of Tac and Resv further suppressed Th17 immune response. In the co-culture study, combination of Resv to Tac significantly decreased HRPTEpiC-induced T cell proliferation compared to Tac alone. Resv treatment in the Jurkat cell induced the expression of AMP-activated protein kinase and suppressed the expression of mammalian target of rapamycin (mTOR), suggesting blocking Th17 pathway by Resv. In the murine skin transplant model, combination of Resv to Tac significantly prolonged skin graft survival accompanied by the suppression of Th17 cells, compared to either the Tac-alone or control groups.

**Conclusion:**

The results of our study suggest that Resv provides additional immunosuppressive effects to Tac by suppressing effector CD4^+^ T cells, especially Th17 cells, in the transplantation setting**.**

## Background

Most cases of allograft rejection in solid organ transplantation (SOT) develop through the activation of allo-immune responses induced by effector CD4^+^ T cells. [1] Out of various CD4^+^ T cell subsets, the activation of Th1 and Th17 cells has most commonly been reported to be associated with the development of allograft rejection in SOT, [2–5] and we also reported that Th17 cells and their associated cytokines play a significant role in the development of acute and chronic allograft rejection. [1, 4–10]

Immune suppressants currently in use mainly take effect by down-regulating the proliferation or differentiation of T cells, [7, 11] and tacrolimus (Tac) is a promising immunosuppressant in regulating T cell responses. Until now, the effect of Tac on regulation of Th17 derived immune cells has been controversial. Previous reports have shown that Tac effectively suppressed Th17 immune responses in experimental models of airway inflammation, osteoclastogenesis and psoriasis, [12–14] but the suppressive effects Tac on Th17 immune responses in kidney transplantation were shown to be somewhat inadequate. [15, 16] Therefore, if a substance that effectively inhibits Th17 immune responses is administered together with Tac the desired immunosuppressive effect may be expected.

In this study, we chose Resveratrol (Resv) as a regulator for Th17 immune responses, combined with Tac. Resv is a natural polyphenol compound and its therapeutic effects have been reported in metabolic disorders, such as diabetic nephropathy. [17] Recent studies have revealed its immune modulating effects in various immune diseases that primarily depend on Th17 immune responses. [18–20] It is also effective modulator of mammalian target of rapamycin (mTOR) signaling, which plays an important role in the regulation of Th17 cell differentiation. [21]

The aim of this study is to investigate the effect of Resv on Th17 immune responses when added to Tac. To this end, we performed an in vitro study using CD4^+^ T cells isolated from peripheral blood mononuclear cells (PBMCs) of healthy volunteers, and human renal proximal tubular epithelial cells (HRPTEpiCs) An in vivo study using a skin allograft mouse model was also carried out.

## Methods

### In vitro and in vivo study design

We designed three separate in vitro experiments and an in vivo study. First, we evaluated the suppressive effects of Resv on the differentiation of CD4^+^ T cells into Th1 (CD4^+^ IFN-γ^+^), Th2 (CD4^+^ IL-4^+^), Th17 (CD4^+^ IL-17^+^), and Treg (CD4^+^ CD25^+^FOXP3^+^) cells out of the total CD4^+^ T cell population using Th0 polarizing conditions and on the differentiation of Th17 cells using Th17 specific polarizing conditions under a Tac present condition using PBMCs isolated from 6 healthy individuals aged 27–40 years. Second, we performed flow cytometry analysis to investigate the suppressive effects of Resv on the proliferation of T cells which were cultured under Th17 polarizing conditions and thereafter activated in a co-culture system with HRPTEpiCs under Tac with or without Resv. Third, we investigated the pathways involved in the suppressive effects of Resv on Th17 cell activation using the Jurkat cell line. Lastly, we conducted an in vivo study using a murine skin transplant model to investigate the suppressive effects of Resv on Th1 and Th17 cells, and to identify the protective effects of Resv against allograft rejection under Tac treatment.

#### Isolation and purification of CD4^+^ T from PBMCs of healthy volunteers

PBMCs were isolated from heparinized blood of healthy volunteers using Ficoll–Hypaque density-gradient centrifugation (GE Healthcare; PA, USA). The isolated cells were cultured as previously described. [22] In brief, a cell suspension of 1 × 10^6^ cells/mL was prepared in RPMI1640 medium supplemented with 10% fetal calf serum (FCS), 100 U/mL penicillin, 100 mg/mL streptomycin, and 2mML-glutamine. CD4^+^ T cells were isolated from the PBMCs using monoclonal anti-human-CD4 antibodies conjugated to microbeads (MicroBeads; Miltenyi Biotech, Bergisch Gladbach, Germany). For cytokine detection at the single-cell level, PBMCs were stimulated with 50 ng/mL phorbol myristate acetate (PMA) (BD Biosciences, San Diego, CA, USA) and 1 μg/mL ionomycin (Sigma, St. Louis, Mo, USA) in the presence of GolgiStop (BD Biosciences, San Diego, CA, USA) for 4 h.

#### Induction of CD4^+^ T cell differentiation using Th0 polarizing condition in vitro

Isolated PBMCs cells (5 × 10^5^) from healthy people were incubated under appropriate conditions for 48 h. To induce CD4^+^ T cell differentiation (Th0 polarizing condition), [15, 16, 23] PBMCs were incubated with anti-CD3 (1 μg/mL) (BD Biosciences, San Diego, CA, USA) and anti-CD28 (1 μg/mL) (BD Biosciences, San Diego, CA, USA). To examine the immunosuppressive effects of Tac (Astellas Pharma Ltd) and Resv (Sigma, St. Louis, Mo, USA), PBMCs were pre-incubated for 1 h with Tac (1, 10 ng/mL) with or without Resv (25, 50 uM), and then stimulated as described above. We compared the differentiation of the T cell subset (Th1, Th17, Th2 and Treg) in nil, Tac (1, 10 ng/mL), and Tac (1 ng/mL) plus Resv (25, 50 uM) conditions using flow cytometry. We also compared cytokine levels (IL-2, IFN-γ, and IL-17) using ELISA in the cell culture supernatant and the expression of mRNA (IFN-γ, and IL-17) using real-time PCR.

#### Induction of Th17 cell differentiation using Th17 polarizing conditions in vitro

Isolated PBMCs cells (5 × 10^5^) from healthy people were incubated under appropriate conditions for 48 h. To induce Th17 differentiation (Th17 polarizing condition), [15, 16] PBMCs (5 × 10^5^) were incubated for 48 h with anti-CD3 (1 μg/mL) (BD Biosciences, San Diego, CA, USA), anti-CD28 (1 μg/mL) (BD Biosciences, San Diego, CA, USA), IL-1β (20 ng/mL) (R&D Systems, Inc. Minneapolis, MN, USA), IL-6 (20 ng/mL) (R&D Systems, Inc. Minneapolis, MN, USA), IL-23 (20 ng/mL) (R&D Systems, Inc. Minneapolis, MN, USA), and interferon-gamma (IFN-γ)-neutralizing antibody (2 μg/mL) (R&D Systems, Inc. Minneapolis, MN, USA) and IL-4-neutralizing antibody (2 μg/mL) (R&D Systems, Inc. Minneapolis, MN, USA). To examine the immunosuppressive effects of Tac (Tacrobell®, Chong Kun Dang Pharma, Seoul, Korea) and Resv (Sigma, St. Louis, Mo, USA), PBMCs were pre-incubated for 1 h with Tac (1, 10 ng/mL) with or without Resv (25, 50 uM), and then stimulated as described above. We compared the differentiation of the Th17 cells in nil, Tac (1, 10 ng/mL), and Tac (1 ng/mL) plus Resv (25, 50 uM) conditions using flow cytometry. We also compared Th17 associated cytokine levels (IL-17, and IL-22) using ELISA in the culture supernatant and the expression of mRNA (IL-17, IL-22) using real-time PCR.

#### Human renal proximal tubular epithelial cells (HRPTEpiCs) and Jurkat cells

HRPTEpiCs were purchased from ScienCell Research Laboratories (ScienCell, San Diego, CA, USA). HRPTEpiCs were maintained in epithelial cell medium (EpiCM, ScienCell, San Diego, CA, USA) supplemented with 2% fetal bovine serum (FBS) at 37 °C with 5% CO_2_. The Jurkat cell lines (ATCC TIB-152, Manassas, VA, USA) were maintained in RPMI 1640 (Gibco Life Technologies, NY, USA) supplemented with 10% FBS at 37 °C with 5% CO_2._

#### PKH-67 labeling of isolated CD4^+^ T cells and co-culture with HRPTEpiCs

All reagents and buffers were at room temperature before starting the PKH-67 labeling step. PKH 67 (Sigma/Aldrich Chemical Company, St. Louis, MO, USA) was diluted according to the manufacturer’s kit directions. PBMCs (3 × 10^6^) were incubated for 48 h under Th17 polarizing conditions as described previously, and then were washed in Dulbecco’s PBS (Gibco/BRL, Grand Island, NY, USA), and were re-suspended in 1 mL of solution C from the kit. The PKH-67 was diluted to 4 × 10^− 6^ M in 1 mL of solution C. The cells were combined with dye, and the tube was inverted several times over 3 mins. About 2 mL of FCS was added to the tube, and it was inverted continuously for 1 min. The cells were then transferred to a 15-mL conical tube with 4 mL of RPMI 1640 without Phenol Red (Sigma/Aldrich Chemical Company, St. Louis, MO, USA), and with 10% FCS (BioWhittaker, Walkersville, MD, USA) and washed three times in the same medium. To examine the immunosuppressive effects of Resv, PKH-67 labeled CD4^+^ T cells were co-cultured with HRPTEpiCs at a ratio of 150,000:20,000 [24] with/without Resv (25, 50 uM) or Tac (1, 10 ng/mL). After 3 days, the harvested cells were examined for proliferation using a FACS Caliber flow cytometer (BD Biosciences, San Diego, CA, USA).

#### Flow cytometry analysis

The cells were surface-stained with CD4-PE/Cy7 (RPA-T4, IgG1; BioLegend, San Diego, CA, USA), and To stain intracellularly, the cells were washed, fixed, permeabilized, and incubated with mAbs against IL-17 (PE, eBio64dec17, IgG1, κ; eBioscience, San Diego, CA, USA), IFN-γ (FITC, 4S.B3, IgG1, κ; eBioscience, San Diego, CA, USA; and PE, B27, IgG1, κ;; Pharmingen, San Diego, CA, USA), IL-4 (APC, MP4-25D2, IgG1, κ; eBioscience, San Diego, CA, USA), and Foxp3 (FITC, PCH101, IgG2a, κ; eBioscience, San Diego, CA, USA). The appropriate isotype controls were used for gating purposes. The cells were analyzed using a FACS Calibur flow cytometer (BD Biosciences, San Diego, CA, USA), and the data were analyzed using the FlowJo software (Tree Star, Ashland, OR, USA).

#### Enzyme-linked immunosorbent assay (ELISA)

IL-2, IFN-γ, IL-17, and IL-22, in the culture supernatants of CD4^+^ T cells were measured using sandwich ELISA (R&D Systems, Inc. Minneapolis, MN, USA), according to the manufacturer’s instructions. The absorbance at 405 nm was measured using an ELISA microplate reader (Molecular Devices).

#### Real-time reverse transcription polymerase chain reaction

mRNA was extracted from CD4^+^ T cells using the TRIzol Reagent (Molecular Research Center, Inc., Cincinnati, OH, USA), according to the manufacturer’s instructions. cDNA was synthesized in a PerkinElmer Cetus DNA thermal cycler (PerkinElmer, Inc., Waltham, MA, USA) using the SuperScript Reverse Transcription system (Takara). A LightCycler 2.0 instrument (Roche Diagnostics; software version 4.0) was used for PCR amplification, and all PCR reactions were performed using LightCycler FastStartDNA Master SYBR Green I (Takara) according to the manufacturer’s instructions. The following primers were used for each molecule: IL-21, 5′ TTC TGC CAG CTC CAG AAG AT 3′(sense) and 5′ TTG TGG AAG GTG GTT TCC TC 3′(antisense); for IFN-γ 5′ TCC CAT GGG TTG TGT GTT TA 3′(sense) and 5′ AAG CAC CAG GCA TGA AAT CT 3′(antisense); for IL-17, 5′ CAA CCG ATC CAC CTC ACC TT 3′(sense) and 5′ GGC ACT TTG CCT CCC AGAT 3′(antisense); and for β-actin, 5′ GGA CTT CGA GCA AGA GAT GG 3′(sense) and 5′ TGT GTT GGG GTA CAG GTC TTTG 3′(antisense). Housekeeping genes (β-actin) were amplified for normalization.

#### Western blot analysis

Jurkat cell lines were pre-incubated for 3 h in the presence of Resv or Tac and then stimulated under Th17 specific polarizing conditions for another 1 h. The membrane was then incubated overnight at 4 °C with primary antibodies against the following: phosphorylated AMPK (p-AMPK), AMPK, phosphorylated mTOR (p-mTOR), mTOR, (all antibodies were from Cell Signaling Technology Inc., Danvers, MA, USA), and β-actin (Sigma, St. Louis, Mo, USA). After washing in TTBS, the reactive bands were visualized using an ECL detection kit and Hyperfilm-ECL reagents (Amersham Pharmacia, Piscataway, NJ, USA).

### In vivo experiment using skin allograft mice model

#### Animals and drugs

All animal experiments were performed in accordance with the Laboratory Animals Welfare Act, the Guide for the Care and Use of Laboratory Animals, and were approved by the Institutional Animal Care and Use Committee at College of Medicine, the Catholic University of Korea (CUMC-2015 − 0057-02).

Eight-week-old male mice weighing 25–30 g (Taconic Anmed, Rockwille, MD, USA) were housed in individual cages in a temperature and light-controlled environment. Tacrolimus (Tacrobell®, Chong Kun Dang Pharma, Seoul, Korea) was diluted in sterile, distilled water to a final concentration of 2 mg/mL. Drug administration started 3 days before skin allograft transplantation. Resveratrol (Sigma, St. Louis, Mo, USA) was dissolved with 0.5% carboxymethyl cellulose sodium salt (CMC). We collected the mouse skin and blood under general anesthesia with 10 mg/kg xylazine hydrochloride (Rompun; Bayer, Leuverkusen, Germany) and 30 mg/kg tiletamine plus zolazepam (Zoletil; Virbac, Carros, France). After which mice were sacrificed by asphyxiation using compressed CO_2_ and chamber.

#### Experimental design

Mice were randomized into three groups, with six animals per group, as follows:

1. Vehicle (VH) group: Daily oral administration of water for 2 weeks.
2. Tac 2 mg/kg group: Daily oral administration of Tac 2 mg/kg for 2 weeks.
3. Tac 2 mg/kg and Resv 100 mg/kg group: Daily oral administration of Tac 2 mg/kg and Resv 100 mg/kg for 2 weeks.

#### Skin allograft operation procedure

Skin allograft operation was carried out as previously described. [25] Briefly, adult male BALB/c (H-2^d^) and C57BL/6 (H-2^b^) mice were used for skin transplantation. Donor and recipients were anesthetized with an intraperitoneal injection. Tail skin of C57BL/6 mice, segmented into 1 × 1 cm^**2**^ pieces were used to replace previously removed BALB/c mouse back skin. The skin graft was sutured with six stitches and was covered with gauze and fixed with a bandage. The bandage was removed on day three, postoperative. From this day, photographs were taken daily with a camera. [26]

#### Ex vivo flow cytometry using isolated mice spleen

At day 6 after skin grafting, single-cell suspensions of the spleens of mice were prepared and stained with fluorescently labeled monoclonal antibodies. Splenocytes were stained with various combinations of fluorochrome conjugated antibodies against CD4 (PerCP-Cyanine5.5, IgG2a κ; eBioscience, San Diego, CA, USA). For intracellular staining, the cells were washed, fixed, permeabilized, and incubated with mAbs against IL-17-FITC, and IFN-γ-PE (all from IgG1, κ; eBioscience, San Diego, CA, USA). The cells were analyzed using a FACS Calibur flow cytometer (BD Biosciences, San Diego, CA, USA). The data were analyzed using the FlowJo software (Tree Star, Ashland, OR, USA).

#### Histology

The transplanted skin graft was removed at 6 days post-transplant in 1 mouse from each group. The skin grafts were fixed in 10% buffered formalin, and tissues were then embedded in paraffin. 4-μm tissue sections were stained with hematoxylin-eosin (H&E) (Sigma, St. Louis, Mo, USA) to assess cellular infiltration.

#### Statistical analysis

Statistical analysis was conducted using the SPSS software (version 16.0; SPSS Inc., Chicago, IL, USA). Continuous variables were summarized as mean ± SD. A non-parametric Wilcoxon signed-rank test was used to compare T cell suppression, cytokine production, and gene expression between the control and treatment groups. Skin allograft survival was compared using the Kaplan-Meier method and Log-rank test**.** A *p* value of < 0.05 was considered to be statistically significant.

## Results

### The suppressive effects of Resv on the differentiation of CD4^+^ T cells into effector T cells

**Fig. 1:**
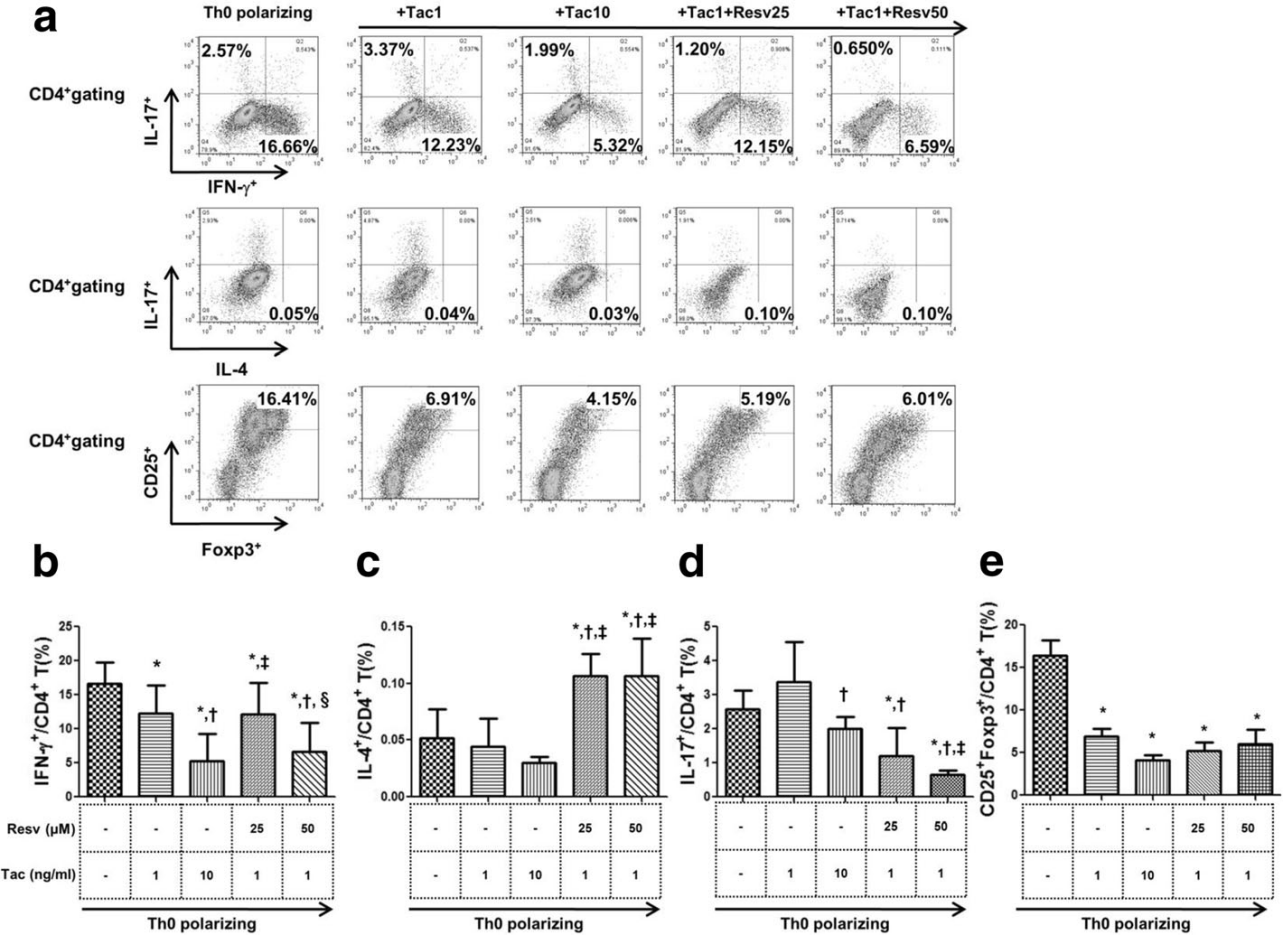
The suppressive effects of Resv on the differentiation of CD4^+^T cells into effector T cells. (**a**) Representative figure of flow cytometry using CD4^+^T stained with anti-IFN-γ FITC, anti-IL-17 PE and anti-IL-4 APC. The effect of Tac with or without Resv on (**b**) Th1 (IFN-γ^+^/CD4^+^ T cells), (**c**) Th2 (IL-4^+^/CD4^+^ T cells) (**d**) Th17 (IL-17^+^/CD4^+^ T cells) and **(e)** Treg (CD25^+^Foxp3^+^/CD4^+^ T cells) under Th0 polarizing conditions. *P < 0.05 vs. Th0 polarizing ^†^P < 0.05 vs. Tac 1 ng/mL group ^‡^P < 0.05 vs. Tac 10 ng/mL group §P < 0.05 vs. Tac 1 ng/mL + Resv 25 μM group. Resv, Resveratrol; Tac, tacrolimus; Treg, regulatory T cells

The proportions of Th1, Th2, Th17, and Treg cells to the total CD4^+^ T cell population in each condition are presented in Fig. 1a. Tac significantly reduced the proportion of Th1 cells in comparison to cells under Th0 polarizing conditions, in a dose dependent manner (*P* < 0.05 for both). Combination of Resv (25, 50 μM) to Tac (1 ng/mL) also showed significant suppressive effects on Th1 cells compared to cells under Th0 polarizing conditions (P < 0.05 for both) (Fig. 1b). The combination of Resv 50 μM to Tac 1 ng/mL, especially displayed stronger significant suppressive effects than the Tac 1 ng/mL alone condition (*P* < 0.05). Treatment with Tac (1, 10 ng/mL) resulted in a decreasing tendency of the proportion of Th2 cells. In contrast, combination of Resv (both 25 and 50 μM) to Tac (1 ng/mL) resulted in a significant increase of the proportion of Th2 cells compared to the Th0 polarizing condition and Tac treatment condition (*P* < 0.05 for all) (Fig. 1c). Treatment with Tac (1 ng/mL) did not suppress Th17 cells in comparison to the Th0 polarizing condition. Combination of Resv (both 25 and 50 μM) to Tac (1 ng/mL), however, resulted in a significant reduction of the proportion of Th17 cells compared to the Th0 polarizing condition alone, in a dose dependent manner (P < 0.05 for both) (Fig. 1d). In regard to Treg cells, Tac significantly decreased the proportion of Treg cells in comparison to the Th0 polarizing condition, and combination of Resv to Tac did not change the proportion of Treg cells (Fig. 1e).

### The suppressive effects of Resv on inflammatory cytokine production from isolated CD4^+^ T cells

**Fig. 2:**
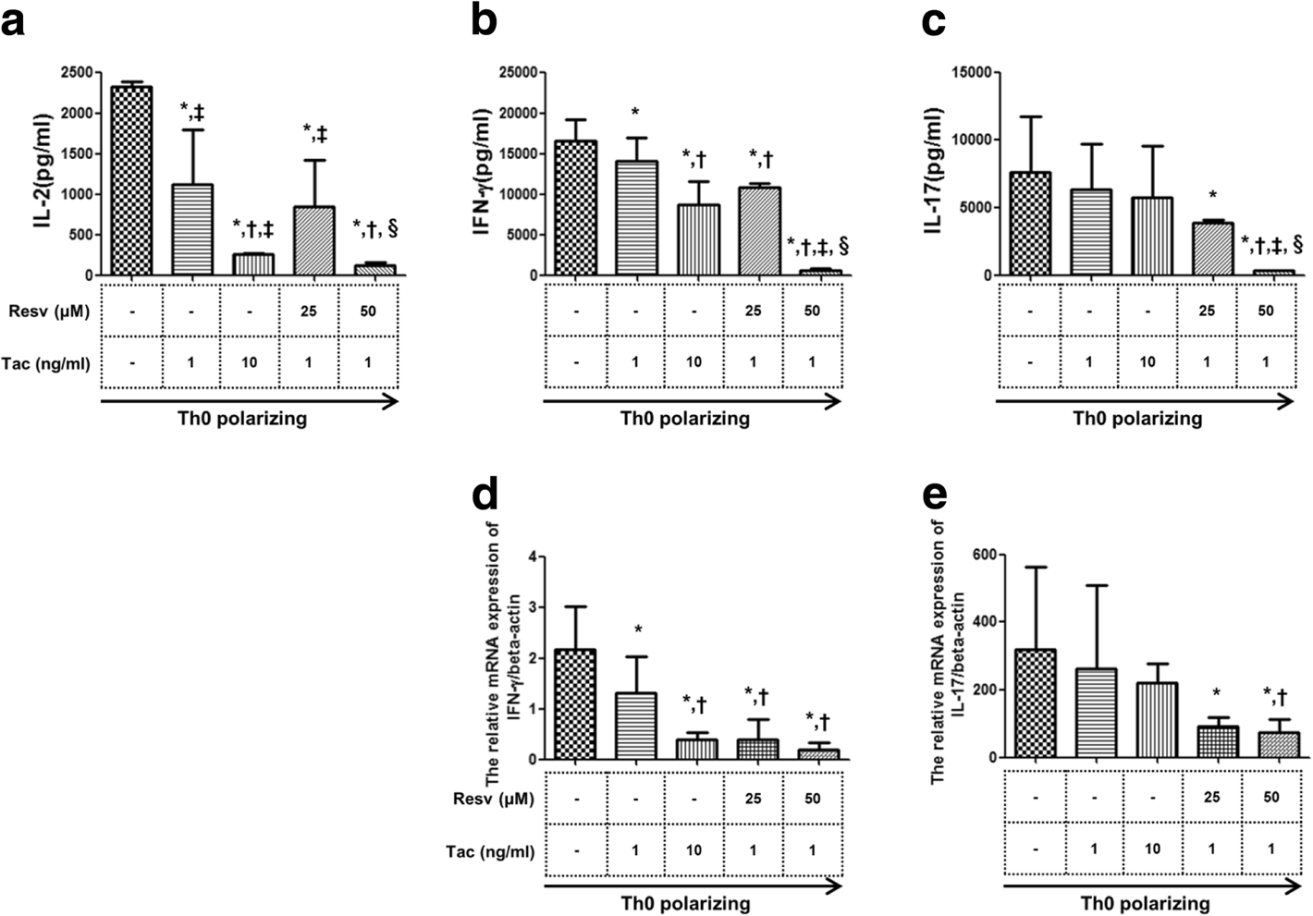
The suppressive effects of Resv on inflammatory cytokine production and mRNA expression. The effects of Tac with or without Resv on the concentration of (**a**) IL-2 (**b**) IFN-γ (**c**) IL-17 in the culture-supernatant and effects on the **e**xpression of (**d**) IFN-γ (**e**) IL-17 mRNA measured using real-time PCR. Bars show the means *P < 0.05 vs. Th0 polarizing ^†^P < 0.05 vs. Tac 1 ng/mL group ^‡^P < 0.05 vs. Tac 10 ng/mL group ^§^P < 0.05 vs. Tac 1 ng/mL + Resv 25 μM group. Resv, Resveratrol; Tac, tacrolimus

Tac (1, 10 ng/mL) suppressed IL-2 levels to a greater extent than the Th0 polarizing conditions in a dose dependent manner (*p* < 0.05 for both). Combination of Resv (25, 50 μM) to Tac (1 ng/mL) also showed significant suppressive effects on the level of IL-2 in a dose dependent manner (*p* < 0.05 for both vs. the Th0 polarizing condition) and combination of Resv (50 μM) to Tac (1 ng/mL) showed even more significant suppressive effects than the Tac (1 ng/mL) alone condition. (Fig. 2a) Tac (1, 10 ng/mL) suppressed IFN-γ levels to a greater extent than the Th0 polarizing condition (*p* < 0.05 for both) and combination of Resv (25, 50 μM) to Tac (1 ng/mL) also significantly decreased IFN-γ level compared to the Th0 polarizing condition or Tac 1 ng/mL alone condition (*p* < 0.05 for all) (Fig. 2b). Both doses of Tac (1, 10 ng/mL) did not decrease the level of IL-17, but combination of Resv (25, 50 μM) to Tac (1 ng/mL) significantly decreased IL-17 compared to the Th0 polarizing condition in a dose dependent manner (*p* < 0.05 for both) (Fig. 2c). In all treatment conditions (Tac alone and Tac plus Resv), the mRNA expression of IFN-γ decreased significantly compared to Th0 polarizing conditions and Resv (50 μM) plus Tac (1 ng/mL) showed particularly more significant suppressive effects than with Tac (1 ng/mL) alone (Fig. 2d). The expression of IL-17 mRNA was not significantly suppressed by Tac alone. But, the combination of Resv (25, 50 μM + Tac 1 ng/mL) significantly suppressed the expression of IL-17 mRNA compared to the Th0 polarizing condition (*P* < 0.05 for all) (Fig. 2e).

### The suppressive effects of Resv on the differentiation of Th17 cells under Th17 specific polarizing conditions

**Fig. 3:**
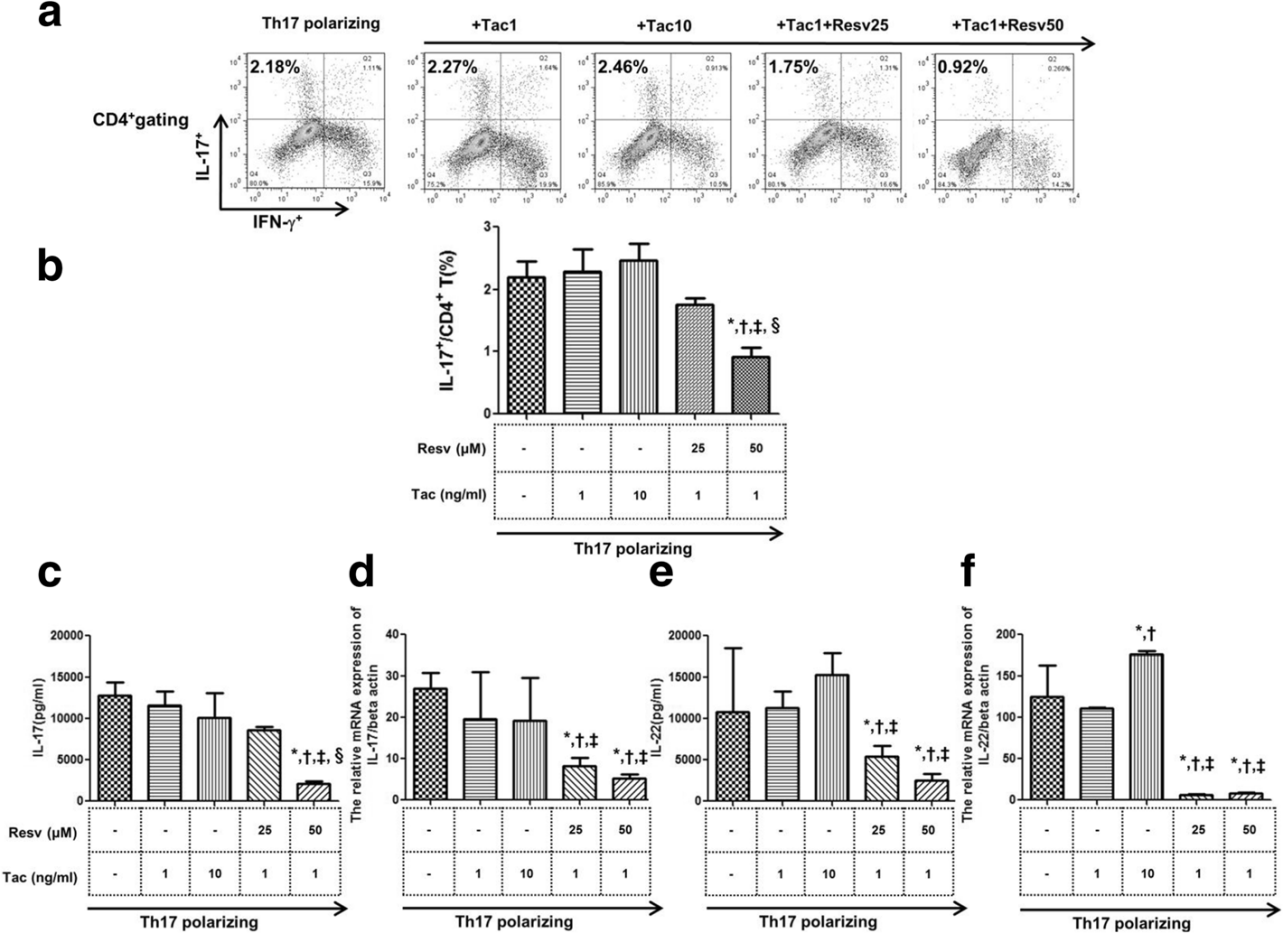
The suppressive effects of Resv on the differentiation of Th17 cells under Th17 specific polarizing conditions. Effects of Tac with or without Resv on CD4^+^ T cells isolated from PBMCs of healthy donors and cultured under Th17 polarizing conditions. (**a**) The percentage of CD4^+^ T cells producing IL-17 was measured by flow cytometry. (**b**) The effects of Tac with or without Resv on IL-17^+^ /CD4^+^ T cells, and effects on (**c**) IL-17 levels (or (**e**) IL-22) and on the expression of (**d**) IL-17 (or (**f**) IL-22) mRNA. *P < 0.05 vs. Th17 polarizing ^†^P < 0.05 vs. Tac 1 ng/mL group ^‡^P < 0.05 vs. Tac 10 ng/mL group ^§^P < 0.05 vs. Tac 1 ng/mL + Resv 25 μM group. Resv, Resveratrol; Tac, tacrolimus

Pre-incubation of PBMCs isolated from 6 healthy donors with Tac (1, 10 ng/mL) did not suppress the proportion of Th17 cells compared to cells under Th17 polarizing conditions alone. However, the combined use of Resv (50 nM) to Tac (1 ng/mL) resulted in a significant reduction of Th17 cell proportions compared to Th17 polarizing conditions, and Tac 1 or 10 ng/mL conditions (*P* < 0.05 for all) (Fig. 3b). Combination of Resv (50 μM) to Tac (1 ng/mL) also significantly decreased IL-17 compared to the Th17 polarizing condition and Tac alone (1, 10 mg/mL) (P < 0.05 for each) (Fig. 3c). Pre-incubation with Tac (1, 10 ng/mL) did not suppress IL-17 mRNA expression compared to the Th17 control, but combination of Resv (25, 50 uM) to Tac (1 ng/mL) significantly suppressed IL-17 mRNA compared to both the Th17 control and Tac alone (1, 10 ng/mL) (Fig. 3d). The same was true for IL-22 protein levels and mRNA expression as well. Tac alone (1, 10 ng/mL) did not affect IL-22 protein levels or expression of IL-22 mRNA compared to the Th17 polarizing condition. But combination of Resv (25, 50 μM) to Tac (1 ng/mL) significantly decreased IL-22 protein levels (P < 0.05 for all) and also mRNA expression of IL-22 compared to the Th17 polarizing condition or Tac alone (1, 10 ng/mL) condition (Fig. 3e and f).

### The suppressive effects of Resv on the proliferation of CD4^+^ T cells after co-culture with HRPTEpiCs

**Fig. 4:**
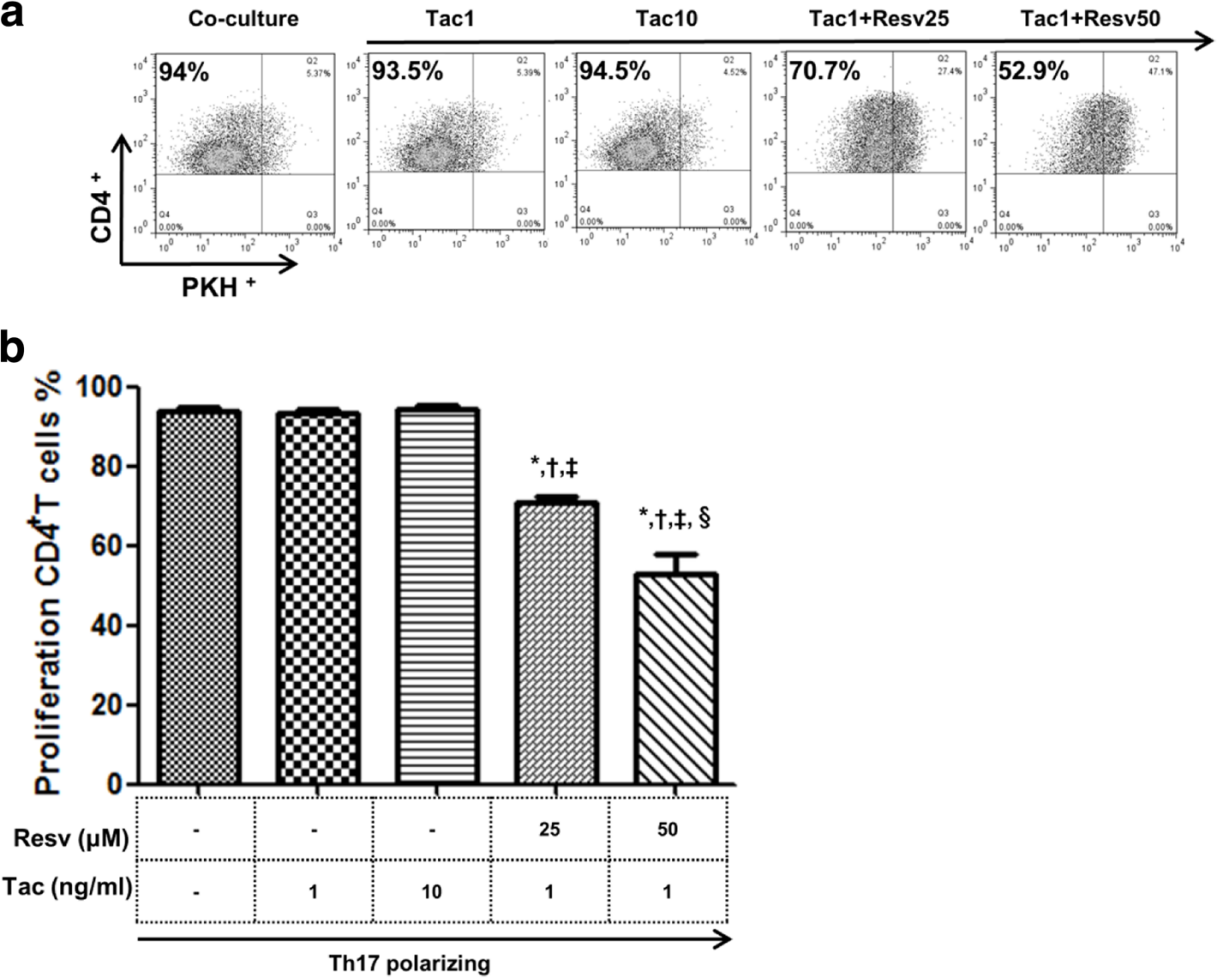
Suppressive effects of Resv on HRPTEpiC-reactive proliferation of CD4^+^ PKH^−^T cells. We co-cultured PKH-67 labeled T cells with HRPTEpiCs and analyzed the differentiation of T cells by counting PKH (−) T cells. (**a**) The percentage of target cells was measured via flow cytometry. (**b**) The proportion (%) of proliferating CD4^+^ T cells. Bars show means. *P < 0.05 vs. Th17 polarizing condition ^†^P < 0.05 vs. Tac 1 ng/mL group ^‡^P < 0.05 vs. Tac 1 ng/mL group ^§^P < 0.05 vs. Tac 1 ng/mL + Resv 25 μM group. Resv, Resveratrol; Tac, tacrolimus

We measured HRPTEpiC-reactive CD4^+^ T cell proliferation using PKH-labeled CD4^+^ T cells in the presence Tac, with or without Resv (25, 50 μM). PBMCs (3 × 10^6^) were incubated for 48 h under Th17 polarizing conditions and then were co-cultured with HRPTEpiCs for 3 days. As a result, Tac (1, 10 ng/mL) did not inhibit the proliferation of HRPTEpiC-reactive CD4^+^ T cells (PKH^−^CD4^+^ T) compared to the control. However, combination of Resv (25, 50 μM) to Tac (1 ng/mL) significantly reduced the proportion of PKH^−^CD4^+^ T cells compared to the control and also the Tac alone condition (1, 10 ng/mL) (*p* < 0.05 for all) (Fig. 4b).

### The pathways involved in the suppressive effects of Resv on the differentiation of CD4^+^ T cells

**Fig. 5:**
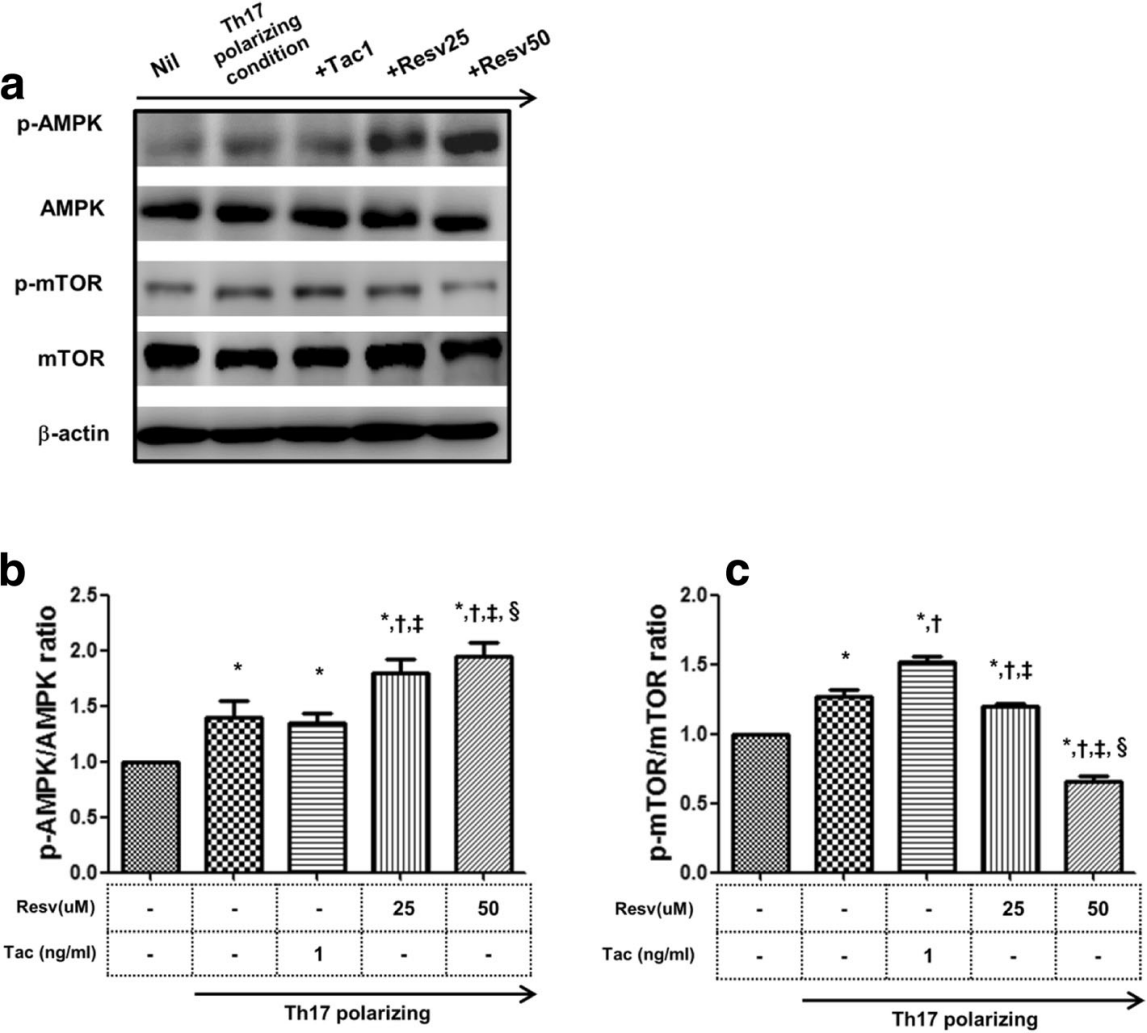
Effects of Tac or Rasv on the expression of mTOR and AMPK proteins in the Jurkat cell line. (**a**) Immunoblotting of p-Ampk, Ampk, p-mTOR,, and mTOR in the Jurkat cell line pretreated with Tac (1 ng/mL) or Resv (25, 50 nM) and then cultured under Th17-differentiation conditions. Stimulation of Jurkat cells under Th17-differentiation conditions activated phosphorylation of (**b**) AMPK, and (**c**) mTOR as detected by Western blotting and shown by the ratio of phosphorylated proteins to total proteins. Tac did not change p-AMPK or p-mTOR. In contrast, treatment with Resv resulted in the reduction of p-mTOR and an increase in p-AMPK. *P < 0.05 vs. Nil and ^†^P < 0.05 vs. Th17 polarizing condition and ^‡^P < 0.05 vs. Tac 1 ng/mL. Resv, Resveratrol; Tac, tacrolimus

Using the Jurkat cell line, we investigated the molecular mechanisms modulated by Resv in activated CD4^+^ T cells under Th17 polarizing conditions, especially focusing on the mTOR/AMPK signaling pathway. As shown in Fig. 5, the level of p-mTOR and p-AMPK increased significantly under the Th17 polarizing condition compared to the Nil condition (139 ± 14% [p-mTOR], 127 ± 5% [p-AMPK] vs. 100%, *p* < 0.05 for both) (Fig. 5a). Tac (1 ng/mL) did not affect the level of both p-mTOR and p-AMPK. In contrast, Resv (25, 50 μM) significantly increased the level of p-AMPK in terms of relative intensity compared to the Th17 polarizing condition and also compared to the Tac alone condition (1 ng/mL)(*p* < 0.05 for each)(Fig. 5b). In addition, Resv 50 μM significantly decreased the levels of p-mTOR in terms of relative intensity compared to the Th17 polarizing condition and also compared to the Tac alone condition (p < 0.05 for each) (Fig. 5c).

### The effects of Resv on allograft survival in the murine skin transplant model under tac treatment

**Fig. 6:**
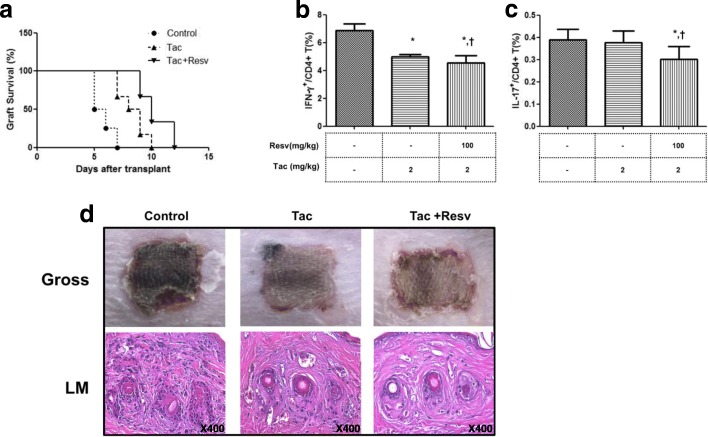
Skin allograft survival in mice treated with Resv and Tac. (**a**) Kaplan-Meier Survival curve of skin allografts in each group with six animals per group. (**b**, **c**) The proportion (%) of IFN-γ^+^/CD4^+^ T cells, and IL-17^+^/CD4^+^ T cells in isolated spleen cells from each group was measured via flow cytometry in six animals per group. (**d**) Histological changes in skin allografts at day 6. The inflammatory cell infiltration of the skin allograft was significantly reduced in the Tac alone group compared to the control group. Combined use of Tac and Resv further decreased inflammatory cell infiltration in skin graft tissue (Magnification × 400). *P < 0.05 vs Control ^†^P < 0.05 vs. Tac 2 mg/kg group. Resv, Resveratrol; Tac, tacrolimus

Skin allografts in non-treatment mice were rejected with a median survival time of 5.7 ± 0.88 days (Fig. 6a). However, treatment with Tac (2 mg/kg) significantly prolonged the survival after skin allograft (8.3 ± 1.12 days, *p* < 0.05 vs. control). Interestingly, the group injected with Tac and Resv (100 mg/kg) showed greater prolongation of graft survival compared to the control or Tac alone groups (10.3 ± 1.36 days, p < 0.05 vs. control or Tac alone). An ex vivo analysis using mouse spleen cells isolated on day 6 after the skin graft showed that the use of Tac (2 mg/kg) resulted in a significant reduction of Th1 cells (p < 0.05 vs. control) but not in reduction of Th17 cells. Combined use of Tac and Resv significantly reduced Th1 compared to the control (*P* < 0.05) and also reduced Th17 cells in comparison with both the control and Tac alone groups (*P* < 0.05 for both) (Fig. 6b and c). Six days after transplantation, the graft was removed from the recipients for pathologic examination. The inflammatory cell infiltration of the skin allograft was significantly reduced in the Tac alone group compared to the control group, and the combined use of Tac and Resv further decreased inflammatory cell infiltration of the skin graft tissue (Fig. 6d).

## Discussion

The results of our study demonstrate that combination of Resv to Tac is effective in regulating CD4^+^ T cells, including Th17 cells under Tac present conditions. An in vitro study using PBMCs and HRPTEpiCs, indicates that the combination of Resv to Tac more effectively suppressed the differentiation of Th17 cells and production of associated cytokines compared to the Tac alone condition. Furthermore, in our in vivo experiment using the murine skin transplant model, combination of Resv to Tac significantly improved skin allograft survival compared to that of the group with Tac treatment alone, with effective suppression of Th17 cells.

The effects of Tac on regulation of Th17 derived immune cells is controversial. The suppressive effects of Tac on Th17 immune responses has been reported in various experimental studies such as airway inflammation, osteoclastogenesis and psoriasis. [12–14] On the other hand, there are only a small number of animal studies on the effects of calcineurin inhibitors (CNIs) on Th17 responses in transplantation. We previously reported that Tac failed to suppress IL-17 immune responses in kidney transplantation recipients. [15, 16] In this study, we confirmed that Tac alone is not effective in suppressing Th17 immune response. The reason for this is related to the Tac dose. We used lower Tac doses (1 ng/ml, 10 ng/ml) compared to other experimental studies, based on a cytoxicity test. Higher doses of Tac decreased viability of PBMCs, thus, we chose Tac doses without significant cytoxicity. Furthermore, we considered therapeutic levels used in clinical practice.

First, we intended to demonstrate the effects of Resv on the differentiation of CD4^+^ T cells in the presence of Tac using a well-established in vitro study model. [16, 27] Tac is well known to be very effective in the suppression of most effector CD4^+^ T cell subsets. [28–30] However, we previously found that the effect of Tac on the Th17 is relatively limited because this drug mainly targets the Th1 pathway. [31, 32] As expected, in the Tac alone condition, Th1, Th2 and Treg were significantly suppressed, but effects on Th17 cells were limited. When we combination Resv to Tac, Th17 cells were significantly suppressed compared to Tac alone, which suggests compensatory effects of Resv on Tac. Meanwhile, Resv increased the proportion of Th2 cells, and these observations are consistent with those of previous studies that showed that Resv suppressed the overall differentiation of T cells and resulted in a shift from the Th1 to the Th2 phenotype. [33, 34] In this study, Resv failed to increase the proportion of Tregs which was decreased by Tac. The reason is unclear, but previous studies showed contradictory results in regard to the effect of Resv on Tregs. [35–37]. Another possible reason may be that Resv did not overcome the suppressive effect of Tac on Tregs, but further studies are required to clarify this issue.

We also investigated the changes in T cell associated cytokine levels, including IL-2, IFN-γ and IL-17, by Tac with or without Resv using ELISA. Tac significantly suppressed IL-2, a representative T cell cytokine, as well as IFN-γ, the Th1 cytokine, but it did not decrease IL-17 levels. [38] Combination of Resv to Tac suppressed all of IL-2, IFN-γ and also effectively suppressed IL-17 compared to the Tac alone condition as shown by our flowcytometry results. In real time PCR for mRNA expression analysis, IFN-γ expression was significantly suppressed by Tac and further suppressed by Resv. IL-17 expression was not suppressed by Tac alone, but it was significantly suppressed by the combination of Resv to Tac. When we used Th17 polarizing conditions to focus on the effects of Resv on Th17 cells, the same was true for Th17 differentiation, Th17 cytokine and mRNA expression. Tac alone did not show significant effects on the Th17 pathway under the Th17 polarizing condition. However, combination of Resv to Tac effectively suppressed Th17 cells, levels of IL-17and IL-22 proteins and also the expression of IL-17 and IL-22 mRNA. These results are consistent with prior reports that Resv suppresses effector T cell differentiation and also affects T cell maturation by suppressing the proliferation of the inflammatory Th17 phenotype. [35]

Next, we investigated the effects of Resv on CD4^+^ T cell differentiation in an in vitro model, mimicing organ transplantation. The differentiation pattern of T cells in the allograft during TCMR may be different from that observed in peripheral blood. Therefore, we used tubular epithelial cells as key targets of the allo-reactive T cells. [39, 40] After contact with HRPTEpiFCs, a significant proliferation of the CD4^+^ T cells was detected. The added of Tac alone did not suppress the proliferation of CD4^+^ T cells. However, the combination of Resv to Tac significantly diminished the proliferation of CD4^+^ T cells compared to activated CD4^+^ T cell conditions or Tac alone. Unfortunately, we did not inspect the subset of proliferating CD4^+^ T cells. We cultured isolated PBMCs under Th17 polarizing conditions before PKH-labeling, hence the proportion of Th17 cells in PKH-labeled T cells may be higher than that of un-stimulated PBMCs. Therefore, it is possible that Tac alone may not be effective in suppressing the PKH^−^CD4^+^ T cells as demonstrated in our in vitro study using PBMCs. Another possible reason is that since almost all T–cell subsets are actively proliferated after contact with HRPTEpiCs, including an increase in the memory T cell subset, this may result in resistance to Tac-based immunosuppresison in the in vitro study. [41] More effective immune suppression by the combination of Resv to Tac may result in the suppression of the proliferating memory T cell subtypes. However, further investigation may be required to clarify this issue.

Recent studies have shown that the mTOR/AMPK pathway plays an essential role in the differentiation of T cells. [42] mTOR is activated upon stimulation of the T cell receptor, and it plays an important role in the regulation of Th17 differentiation. [21] In addition, the activation of AMP-activated protein kinase (AMPK) can diminish mTOR signaling through the phosphorylation of TSC2 and RAPTOR, which is a crucial component of mTORC1. [43] Based on the above findings**,** we investigated whether the AMPK/mTOR pathway is involved in the suppressive effects of Resv on T cells using the Jurkat cell line. In our study, Tac alone shows an insignificant effect on the phosphorylation of AMPK or mTOR pathway. However, in the Resv condition, a significant increase in the phosphorylation of AMPK and a reciprocal decrease in the phosphorylation of mTOR was detected in a dose-dependent manner. This result suggests that AMPK/mTOR signaling may be involved in the suppressive effects of Resv on CD4^+^ T cell proliferation. [19, 44, 45]

Finally, we intended to investigate the immune modulating effects of Resv using a well-established skin allograft mouse model. [46] The use of Tac significantly improved skin allograft survival when compared to the control group, and this result is similar to the survival rate of previous skin allograft mouse models. [47] The combination of Resv to Tac further prolonged survival duration of skin allograft. In an ex vivo study using mice spleen cells, the proportion of Th1 cells were significantly decreased in the group with Tac alone, but Th17 was not suppressed in this group. However, the combination of Resv to Tac resulted in a significant decrease in the proportion of Th17 cells, which is consistent with our in vitro studies. Many studies have demonstrated that acute rejection of allografts is associated with an increase in Th1 reactivity, with high levels of IL-2, IFN-γ mRNA and protein detected within the grafts. [46] Also, blocking the IL-17 function in a rat cardiac allograft transplantation model produced a significant increase in graft survival. The same group later showed that IL-17 antagonism inhibited rejection in a murine aortic transplantation model. [48] Therefore, it is possible that effective regulation of both Th1 and Th17 through the combination of Resv to Tac may result in better allograft survival.

A limitation of our experiment is that the experimental concentration of Resv is higher than a realistic dose. Hence, we could not suggest an appropriate dose of Resv that may show immunologic benefits in clinical practice. However, Resv contained in various healthy foods has shown significant health benefits in the general population [49, 50]. Therefore it is possible that an ordinary dose of Resv taken in such healthy foods may produce the immunologic benefits shown in this study. Second, another mechanism of Resv can be associated with the effective suppression of Th17 in this study. For example, Resv can inhibit CYP enzyme involved in the Tac metabolism. It may result in the increased immune suppressive effect of Tac. [51] However, further translational or clinical research may be required to clarify this issue.

## Conclusions

We found that the combination of Resv to Tac resulted in significant suppressive effects on the differentiation of Th17 cells that are involved in the development of allograft rejection in an in vitro and in vivo study. Therefore, we suggest that Resv can be used as a therapeutic agent to complement the effects of Tac-based immunosuppression in organ transplantation.

## Abbreviations

AMPK: AMP-activated protein kinase
CNIs: calcineurin inhibitors; FCS: fetal calf serum
FBS: fetal bovine serum
H&E: hematoxylin-eosin
HRPTEpiCs: human renal proximal tubular epithelial cells
mTOR: mammalian target of rapamycin
PBMCs: peripheral blood mononuclear cells
Resv: Resveratrol
SOT: solid organ transplantation
TAC: tacrolimus
Th17: T helper 17 cells
